# International practice patterns of dyslipidemia management in patients with chronic kidney disease under nephrology care: is it time to review guideline recommendations?

**DOI:** 10.1186/s12944-023-01833-z

**Published:** 2023-05-25

**Authors:** Viviane Calice-Silva, Daniel Muenz, Michelle M. Y. Wong, Keith McCullough, David Charytan, Helmut Reichel, Bruce Robinson, Benedicte Stengel, Ziad A. Massy, Roberto Pecoits-Filho, on behalf of CKDopps Investigators, Antonio Lopes, Christian Combe, Christian Jacquelinet, Ziad Massy, Johannes Duttlinger, Danilo Fliser, Gerhard Lonnemann, Takashi Wada, Kunihiro Yamagata, Ron Pisoni, Viviane Calice-Silva, Ricardo Sesso, Elodie Speyer, Natalia Alencar de Pinho, Koichi Asahi, Junichi Hoshino, Ichiei Narita, Rachel Perlman, Friedrich Port, Nidhi Sukul, Michelle Wong, Eric Young, Jarcy Zee

**Affiliations:** 1 Pro-Kidney Foundation, Joinville, Brazil; 2 University of Joinville’s Region - UNIVILLE, Joinville, Brazil; 3 Division of Nephrology, Department of Internal Medicine, University of Michigan Medical School, 3989 Research Park Dr, Ann Arbor, MI 48108 USA; 4 Division of Nephrology, Department of Medicine, University of British Columbia, Vancouver, BC Canada; 5 Nephrology Division, New York University Grossman School of Medicine, New York, NY USA; 6 Nephrological Center Villingen-Schwenningen, Villingen-Schwenningen, Germany; 7 Université Paris Saclay, Université Versailles Saint-Quentin en Yvelines, Institut National de La Santé Et de La Recherche Médicale (Inserm), Villejuif, France; 8 Centre de Recherche en Epidémiologie Et Santé Des Populations (CESP), Equipe Epidémiologie Clinique, Villejuif, France; 9 Department of Nephrology, CHU Ambroise Paré, APHP, Boulogne, France

**Keywords:** Chronic kidney disease, Dyslipidemia, Lipids management, LDL-C, Statins

## Abstract

**Background:**

In contrast to guidelines related to lipid therapy in other areas, 2012 Kidney Disease Improving Global Outcomes (KDIGO) guidelines recommend conducting a lipid profile upon diagnosis of chronic kidney disease (CKD) and treating all patients older than 50 years without defining a target for lipid levels. We evaluated multinational practice patterns for lipid management in patients with advanced CKD under nephrology care.

**Methods:**

We analyzed lipid-lowering therapy (LLT), LDL- cholesterol (LDL-C) levels, and nephrologist-specified LDL-C goal upper limits in adult patients with eGFR < 60 ml/min from nephrology clinics in Brazil, France, Germany, and the United States (2014–2019). Models were adjusted for CKD stage, country, cardiovascular risk indicators, sex, and age.

**Results:**

LLT treatment differed significantly by country, from 51% in Germany to 61% in the US and France (*p *= 0.002) for statin monotherapy. For ezetimibe with or without statins, the prevalence was 0.3% in Brazil to 9% in France (< 0.001). Compared with patients not taking lipid-lowering therapy, LDL-C was lower among treated patients (*p* < 0.0001) and differed significantly by country (*p* < 0.0001). At the patient level, the LDL-C levels and statin prescription did not vary significantly by CKD stage (*p* = 0.09 LDL-C and *p* = 0.24 statin use). Between 7—23% of untreated patients in each country had LDL-C ≥ 160 mg/dL. Only 7–17% of nephrologists believed that LDL-C should be < 70 mg/dL.

**Conclusion:**

There is substantial variation in practice patterns regarding LLT across countries but not across CKD stages. Treated patients appear to benefit from LDL-C lowering, yet a significant proportion of hyperlipidemia patients under nephrologist care are not receiving treatment.

**Supplementary Information:**

The online version contains supplementary material available at 10.1186/s12944-023-01833-z.

## Introduction

Patients with chronic kidney disease (CKD) have an extremely high cardiovascular disease (CVD) burden, which increases as CKD progresses. CKD may represent the kidney manifestation of the systemic impact of vascular disease under the influence of exposure to risk factors such as dyslipidemia. Despite the lack of evidence of benefit from lipid-lowering therapies (LLT) to reduce the progression of CKD, dyslipidemia is considered a modifiable CVD risk factor in this high-risk population [1, 2], and LLT has been shown to reduce the risk of atherosclerotic cardiovascular events [3].

Based on the analysis of the evidence specific to CKD patients*, the 2012 Kidney Disease: Improving Global Outcomes (KDIGO) Clinical Practice Guideline for Lipid Management in CKD* [4] recommended conducting a lipid profile upon diagnosis for CKD to establish the diagnosis of severe hypercholesterolemia and/or hypertriglyceridemia and potentially rule out a remediable (secondary) cause if present. These global nephrology guidelines also recommend that all CKD patients ≥ 50 years, and high-risk younger adult patients, should be treated with a statin with or without ezetimibe regardless of lipid levels. In addition, the guidelines did not recommend a follow-up measurement of lipid levels for most patients. These recommendations were based on the results of several clinical studies but principally the SHARP trial results and two meta-analyses [3].

By contrast, different cardiology society guidelines worldwide have provided LDL-cholesterol (LDL-C) targets for CKD patients according to CKD stages and cardiovascular (CV) patients’ risk, which range from < 55 mg/dL for those considered very high risk (CKD stage 4–5) moving to < 70 mg/dL for those at high risk (CKD 3a-3b) and rising to a maximum of 189 mg/dL for those CKD patients between 40–75 years old and with a 10-year atherosclerotic CVD risk of ≥ 7.5% [5, 6]. However, international variations in practice patterns and adherence to these guidelines have not been described until the present.

To evaluate the implementation of these recommendations in real-world clinical practice, we aimed to assess current practice patterns for lipid management in an international cohort of non-dialysis CKD patients under nephrology care. Objectives include describing the prevalence and intensity of statin/ezetimibe prescription, achieved levels of LDL-C, and clinicians’ perceptions of LDL-C goals.

## Materials and methods

### Aim, design, and study setting

With this multinational cross-sectional analysis of baseline data from the Chronic Kidney Disease Outcomes and Practice Patterns Study (CKDopps), we aimed to demonstrate the variation between clinical practices regarding lipid management in CKD-non-dialysis patients, including prescription patterns, achieved LDL-C levels, and nephrologists’ targets for LDL-C.

### Data source

The CKDopps is an ongoing prospective cohort study of Stage 3–5 CKD (eGFR < 60 ml/min) patients treated in nephrologist-led CKD clinics in Brazil, France, Germany, and the United States (US) (2013–2019). Unfortunately, data from Japan were unavailable at the time of this analysis. CKDopps sites were randomly selected from CKD clinics after stratification by region and clinic profile (academic vs. private). The criteria used for clinic selection regarding the geographic region, key clinic characteristics, inclusion and exclusion criteria, and study design, details, and objectives have previously been published [7]. No clinical data were collected beyond those performed as part of usual care, as the aim is to evaluate standard nephrology clinic practices. One exception was laboratory measurements in France, where a standard set of urine and blood tests was requested at baseline, including lipids. CKDopps was approved by national and/or local ethics committees, and patient consent was obtained as required by local ethics regulations.

### Cardiovascular disease and lipids therapy stratification

We categorized all cardiovascular diseases as either atherosclerotic CVD (ASCVD) or non-atherosclerotic CVD (referred to as “other CVD”, meaning CVD that is not atherosclerotic). ASCVD was defined by the following diagnoses/events and procedures: angina (stable or unstable), acute myocardial infarction, transient ischemic attack, claudication/rest pain, aortic aneurysm, stroke (ischemic), renal artery stenting and/or angioplasty, cardiac catheterization, coronary angioplasty, coronary bypass graft, carotid endarterectomy, angiogram, arterial bypass surgery, coronary angiogram, percutaneous transluminal angioplasty, and renal angioplasty and/or stenting. Other CVD was defined by the following diagnoses/events and procedures: cardiac arrest/sudden death, congestive heart failure, cardiomyopathy, valvular heart disease, atrial fibrillation, other arrhythmia, pericarditis and/or tamponade, deep vein thrombosis, tachycardia, pulmonary edema due to exogenous fluid, cerebral hemorrhage, ischemic brain damage/anoxic encephalopathy, hemorrhage from a ruptured vascular aneurysm, valve repair or replacement, aortic aneurysm repair, cardioversion, defibrillator placement, pacemaker placement, and pericardial procedure [8]. The composite CV risk is based on comorbidity burden (any history of coronary disease, diabetes, or ischemic stroke) and age. In addition, LLT intensity was categorized into two categories: atorvastatin and rosuvastatin were classified as high intensity, and all other statins were categorized as low intensity: simvastatin, lovastatin, pravastatin, fluvastatin, cerivastatin, and pitavastatin. This classification was chosen due to the lack of statin doses in the CKDopps database. Thus, the definition suggested by most guidelines of considering statin doses to classify them as low vs. moderate vs. high intensity could not be applied here.

### Statistical analysis

We reported the mean or percentages of patient characteristics at enrollment into CKDopps. These are presented for socio-demographics, laboratory values, dyslipidemia prescriptions, and comorbidities, all presented by CKD stage and country. For LDL-C levels, we also presented the results stratified based on a composite measure of CV risk: diabetes, any history of coronary disease, and ischemic stroke and further stratified by age < 50 versus age ≥ 50. The CV risk factors were based on some of the factors listed in the KDIGO recommendations regarding statin use among patients aged 18–49, such as a) known coronary disease (myocardial infarction or coronary revascularization), b) diabetes mellitus, c) prior ischemic stroke and, d) estimated 10-year incidence of coronary death or non-fatal myocardial infarction > 10% [4].

We assessed country-level patterns of care for lipid management, including (a) prevalence and intensity of statin use (high intensity: atorvastatin and rosuvastatin; low intensity: all other statins), (b) frequency of lipid testing, (c) mean LDL-C levels during CKD progression, (d) the distribution of LDL-C by statin use, and (e) nephrologist-reported LDL-C goal upper limits. Models were adjusted for CV risk factor, CKD stage, country, sex, and age. Statins were classified as high intensity (atorvastatin or rosuvastatin) and low intensity (all other types).

Linear and logistic regression models were used to obtain p values for comparisons of LDL-C levels and the prevalence of statin and/or ezetimibe treatments. In addition, comparisons were made between age groups (< or ≥ 50), countries, and CKD stages. Linear regression models were used on the mean LDL-C by treatment (including statin intensity), country, and CKD stage. Models used generalized estimating equations with an exchangeable working correlation structure to account for patient clustering by the clinic.

## Results

We analyzed 8,194 CKD patients in Brazil (912), France (2,969), Germany (2,761), and the US (1,552) (Table 1). The patients were generally similar, but there was some variation by country. Patients from Brazil were usually younger, more often Black, and had more peripheral artery disease. Patients from France had less severe disease (more CKD stage 3 than stage 4), a slightly higher smoking prevalence, and were prescribed ezetimibe more often. Patients from Germany were generally older and had higher HDL-C levels, LDL-C levels, and prevalence of cardiovascular diseases. US patients had lower HDL-C, LDL-C, and hemoglobin levels and a higher prevalence of diabetes. Within each country, the percentage of CKD patients who were female, diabetic, or had high triglyceride levels was higher in stages 4/5 than in stage 3. In contrast, hemoglobin and LDL-C levels tended to be lower in stages 4/5.

**Table 1:** Patient characteristics at enrollment in CKDopps, by country and CKD stage

	**Brazil**		**France**		**Germany**		**US**	
	**CKD Stage**		**CKD Stage**		**CKD Stage**		**CKD Stage**	
**Characteristics**	**3** **(*n* = 278)**	**4/5** **(*n* = 634)**	**3** **(*n* = 1606)**	**4/5** **(*n* = 1363)**	**3** **(*n* = 678)**	**4/5** **(*n* = 2083)**	**3** **(*n* = 445)**	**4/5** **(*n* = 1107)**
**Demographics**								
Age, y	66 ± 15	65 ± 15	66 ± 12	68 ± 14	71 ± 12	73 ± 12	69 ± 12	68 ± 13
< 50	14%	14%	11%	11%	6%	5%	8%	9%
≥ 50	86%	86%	89%	89%	94%	95%	92%	91%
Female	41%	50%	33%	37%	37%	45%	45%	50%
Black race	33%	25%	3%	2%	-	-	23%	20%
Current smoker	8%	6%	12%	12%	-	-	7%	10%
Body mass index, kg/m^2^	27 ± 5	28 ± 6	29 ± 6	29 ± 6	29 ± 5	29 ± 6	31 ± 7	31 ± 7
**Lipid measurements**								
Has baseline total cholesterol^c^	53%	48%	87%	85%	36%	38%	27%	22%
Total cholesterol, mg/dL	179 ± 42	174 ± 46	186 ± 46	184 ± 47	197 ± 45	194 ± 47	165 ± 39	172 ± 47
< 200	72%	72%	63%	67%	56%	57%	81%	75%
200–239	18%	18%	25%	20%	26%	25%	14%	14%
≥ 240	10%	10%	13%	13%	18%	18%	5%	11%
Has baseline LDL-C^c^	40%	33%	83%	80%	27%	28%	27%	22%
LDL-C, mg/dL	97 ± 31	93 ± 33	103 ± 37	100 ± 37	120 ± 36	117 ± 38	90 ± 32	89 ± 34
< 70	18%	28%	21%	23%	7%	11%	28%	31%
70–99	40%	31%	29%	31%	28%	24%	36%	34%
100–129	25%	25%	25%	25%	23%	28%	26%	22%
130–189	17%	16%	24%	20%	38%	34%	9%	13%
≥ 190	0%	0%	1%	1%	3%	3%	1%	0%
Has baseline HDL-C^c^	46%	39%	80%	80%	24%	27%	27%	22%
HDL-C, mg/dL	46 ± 12	43 ± 12	48 ± 13	47 ± 13	49 ± 12	47 ± 12	44 ± 12	44 ± 13
< 40/50^a^	44%	59%	36%	41%	37%	41%	48%	58%
40/50^a^-59	42%	31%	46%	40%	43%	42%	43%	29%
≥ 60	14%	10%	18%	19%	19%	17%	9%	13%
Has baseline triglycerides^c^	52%	43%	85%	84%	33%	36%	26%	21%
Triglycerides, mg/dL	160 ± 86	166 ± 90	157 ± 89	168 ± 92	179 ± 86	183 ± 97	158 ± 88	179 ± 90
< 150	55%	53%	58%	55%	44%	46%	61%	43%
150–199	21%	19%	18%	19%	26%	21%	12%	24%
200–499	24%	27%	22%	25%	30%	31%	26%	33%
≥ 500	1%	1%	1%	1%	0%	1%	1%	0%
**Other labs**								
Creatinine, mg/dL	1.8 ± 0.5	3.2 ± 1.6	1.6 ± 0.3	2.7 ± 0.7	1.6 ± 0.4	2.6 ± 0.6	1.6 ± 0.4	3.0 ± 1.3
Albumin, g/dL	-	-	3.9 ± 0.4	3.8 ± 0.4	4.2 ± 0.6	4.1 ± 0.6	4.0 ± 0.4	3.8 ± 0.5
Hemoglobin, g/dL	13.2 ± 1.8	11.6 ± 1.8	13.4 ± 1.6	12.5 ± 1.6	13.1 ± 1.7	12.1 ± 1.6	12.5 ± 1.8	11.6 ± 1.8
**Dyslipidemia prescriptions, %**								
Statin	58%	58%	58%	60%	55%	49%	60%	61%
Ezetimibe	1%	0%	8%	9%	4%	4%	3%	2%
Fibrate	5%	4%	3%	3%	2%	1%	5%	5%
Niacin	0%	0%	-	-	0%	0%	2%	1%
Omega-3 fatty acid	0%	1%	1%	1%	0%	0%	17%	12%
Bile acid sequestrant	0%	0%	0%	0%	0%	0%	1%	0%
PCSK9 inhibitor	0%	0%	-	-	0%	0%	0%	0%
Any of the above	59%	60%	62%	64%	57%	52%	68%	66%
**Comorbidity history, %**								
CVD	44%	48%	43%	46%	50%	54%	49%	53%
ASCVD	26%	29%	31%	37%	35%	35%	34%	36%
Myocardial infarction	7%	8%	12%	15%	8%	8%	7%	9%
Angina	10%	13%	6%	8%	2%	2%	7%	7%
Ischemic stroke	9%	7%	6%	8%	8%	8%	5%	7%
Transient ischemic attack	2%	2%	3%	4%	1%	1%	3%	4%
Carotid endarterectomy or stenting	0%	1%	2%	2%	0%	0%	3%	3%
Percutaneous coronary intervention	5%	8%	13%	16%	3%	2%	9%	10%
Other CVD	34%	40%	31%	35%	36%	42%	38%	40%
Peripheral artery disease	20%	24%	15%	17%	17%	19%	16%	15%
Diabetes	44%	48%	42%	44%	47%	49%	54%	58%
Hypertension	94%	97%	90%	92%	97%	98%	96%	96%
High CV risk^b^, % high (vs not)	56%	56%	54%	56%	63%	63%	65%	70%

Patient characteristics are reported as % or mean ± standard deviation

Values less than 0.5% were rounded to 0%

^a^HDL-C < 40 mg/dL for men and < 50 mg/dL for women

^b^High CV risk means having a history of coronary disease, diabetes, or ischemic stroke

^c^Requested laboratory measurements per study protocol in France versus routine measurements in other countries. US, United States

**Fig. 1:**
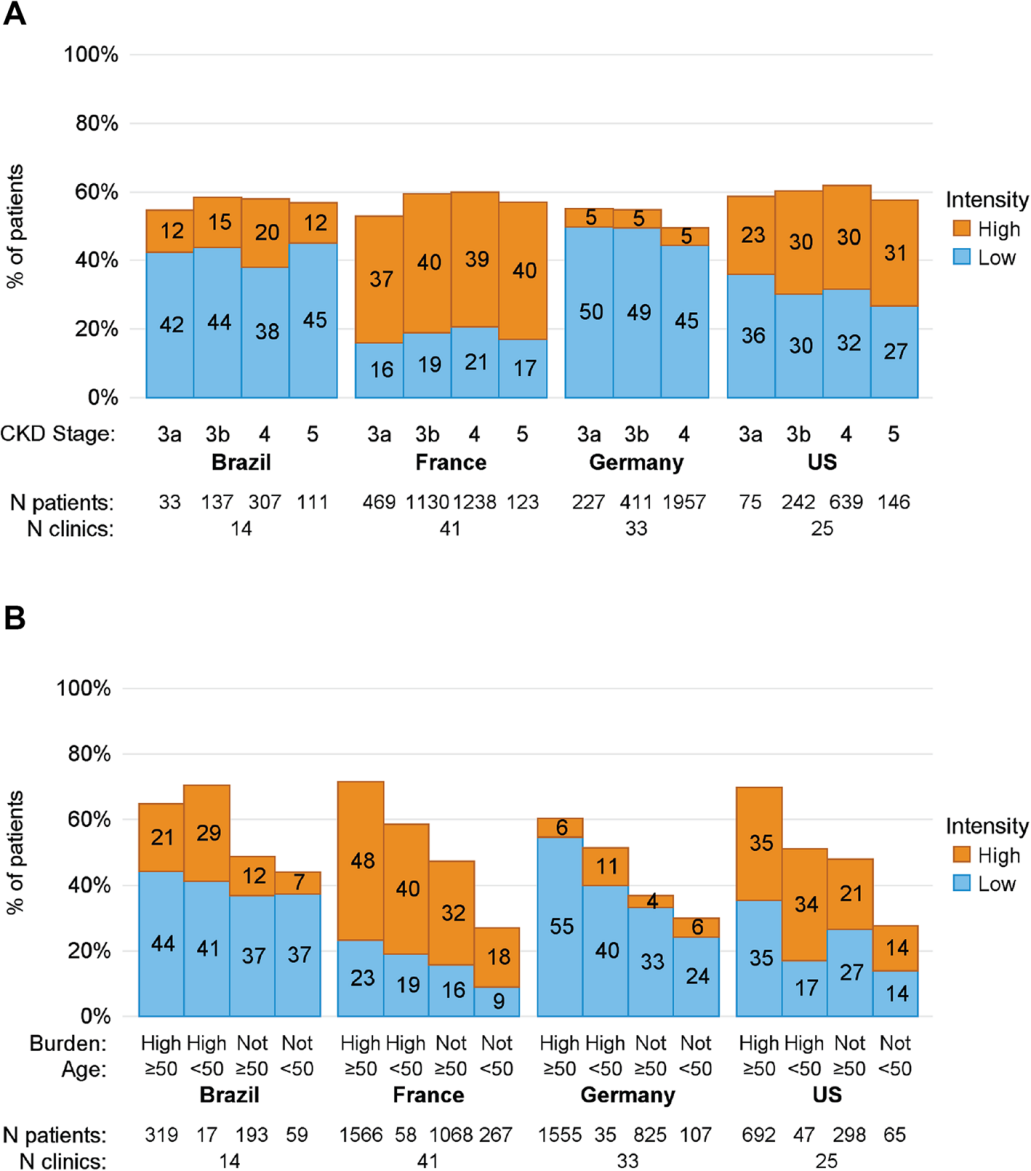
Prevalence and intensity of statin use by country and **A** CKD stage or **B** cardiovascular risk. Atorvastatin and rosuvastatin are categorized as high intensity; all other statins are categorized as low intensity: simvastatin, lovastatin, pravastatin, fluvastatin, cerivastatin, and pitavastatin. The composite cardiovascular (CV) risk is based on comorbidity burden (any history of coronary disease, diabetes, or ischemic stroke) and age

Overall statin use was similar by country and CKD stage, but the types of statins differed between countries (Fig. 1A). Among statin users, patients in France and the US used more high-intensity statins (39% and 30% overall) than patients in Brazil (9%) and Germany (4%). Patients with ASCVD, diabetes, or peripheral artery disease had slightly higher statin use within each country, but there was no consistent pattern in the use of high- versus low-intensity statins between patients with versus without these comorbidities (Supplemental Fig. 1; see file Supplementary Fig. 1). Statin use was higher among patients over the age of 50 compared to patients under the age of 50. Within these age groups, statin use was higher among patients with CV comorbidities or diabetes (Fig. 1B). Outside Germany, where high-intensity statin use was rare, high-intensity statin use was also generally higher among patients aged 50 + and with CV comorbidities.

**Fig. 2:**
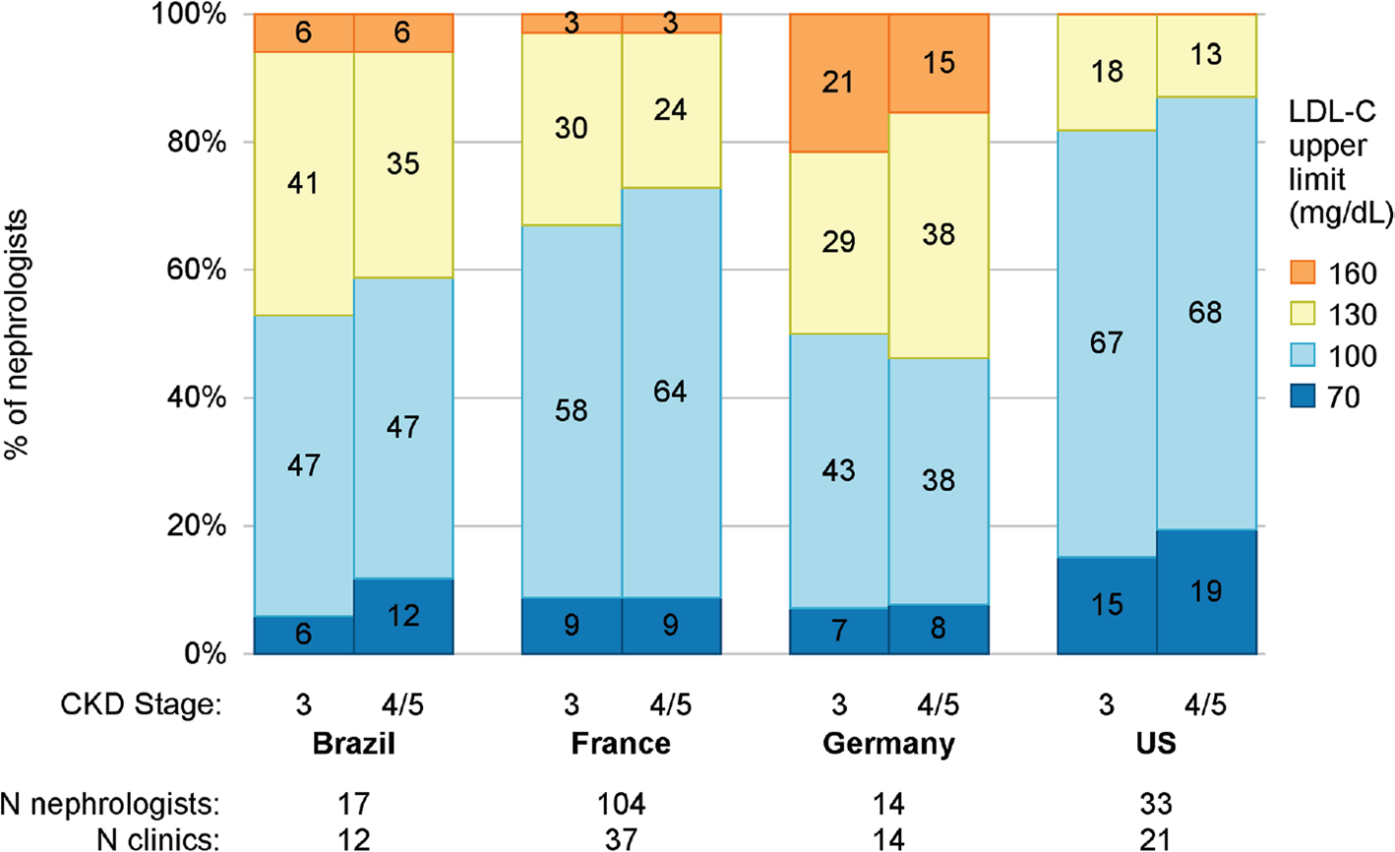
LDL-C (fasting) goal upper limit by country and CKD stage, according to clinic nephrologists. Nephrologists were allowed to respond “No upper limit”; the numbers of nephrologists so responding were 1 (Brazil), 6 (France), 1 (Germany), and 8 (the US)

The upper limit of fasting LDL-C goals by nephrologists varied from country to country (Fig. 2). Consistent with the higher LDL-C levels found among their patients, 50–53% (depending on CKD stage) of the German nephrologists surveyed specified an upper LDL-C level of 130 or 160 mg/dL, compared to 13–18% of US nephrologists, 27–33% of French nephrologists, and 41–47% of Brazilian nephrologists. No US nephrologists specified an upper LDL-C limit of 160; only 3–6% of Brazilian and French nephrologists did, while 15–21% of German nephrologists selected this high level. Only 7–17% of nephrologists believed that LDL-C should be < 70 mg/dL, and 38- 68% would choose LDL < 100 mg/dl as a threshold.

**Fig. 3:**
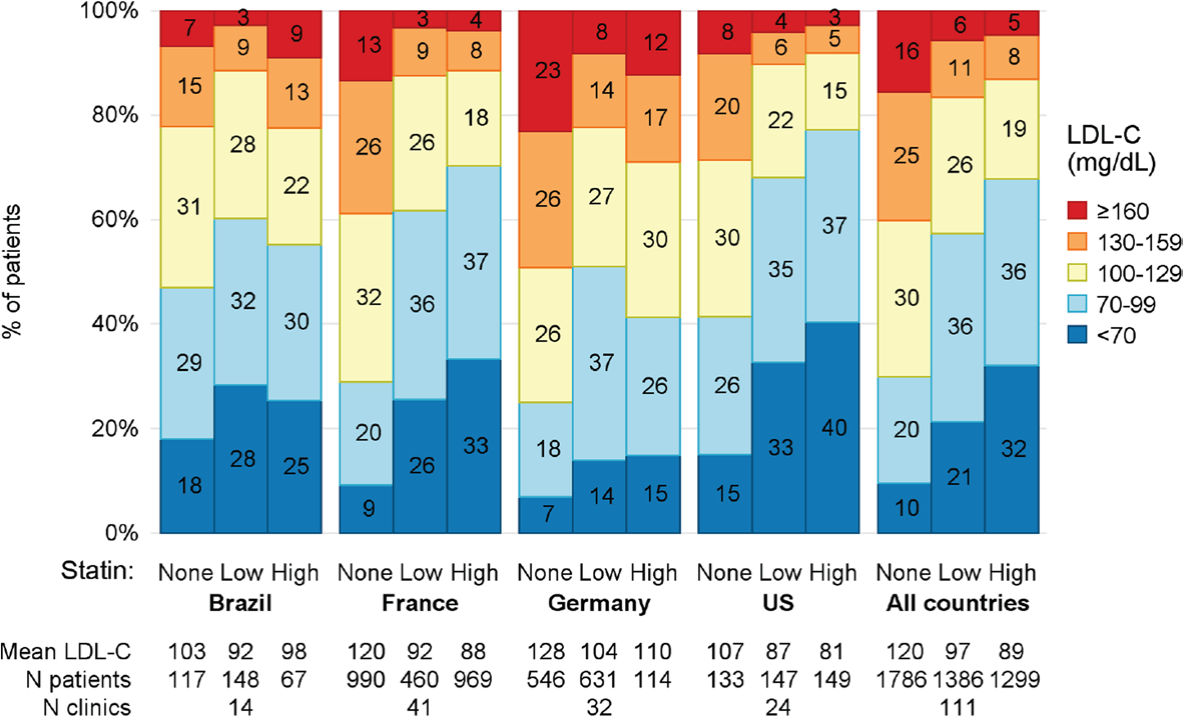
Distribution of LDL-C (mg/dL) by country and statin use and intensity. Statin use was ascertained within 6 months before the LDL-C measurement. To determine statin intensity, patients with no statin use in that time window were categorized as “none”; patients using atorvastatin or rosuvastatin ever within the window were categorized as “high”; and patients using any other statin were categorized as “low”

**Fig. 4:**
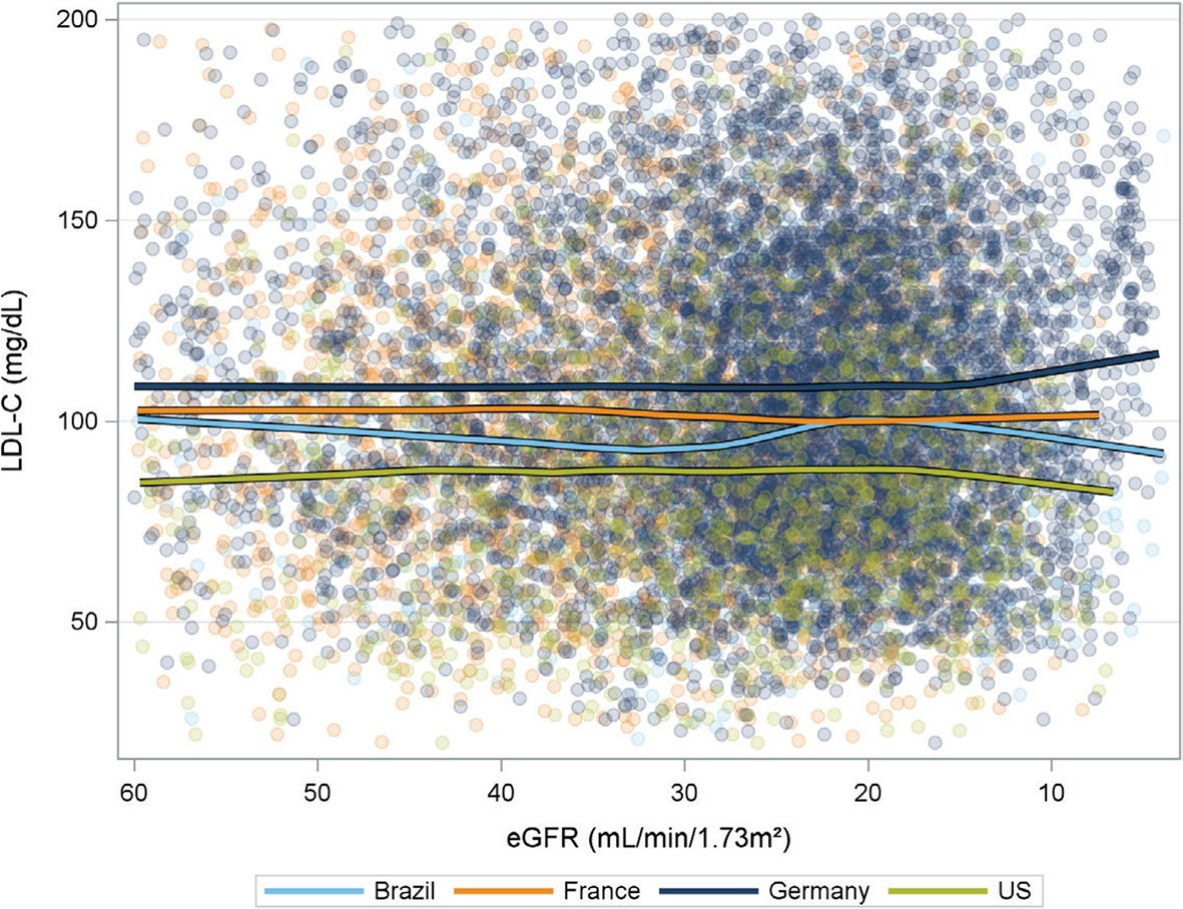
Mean LDL-C levels by eGFR and by country. Representation of Mean LDL-C patients levels considering eGFR and stratified by country

Within each country, the adjusted difference in mean LDL-C between high- and low-intensity statin categories never exceeded 5.5 mg/dL and was statistically significant only in France. In contrast, the combined high and low categories had an adjusted mean LDL-C that was always significantly lower than the “no statin use” category: -7.6 mg/dL for Brazil (*p*-value = 0.019), -25.8 for France (*p*-value < 0.001), -19.9 for Germany (*p*-value < 0.001), and -21.2 for the US (*p*-value < 0.001) (Supplemental Fig. 3; see file Supplementary Fig. 4). The statin intensity-by-country interaction p-value (6 degrees of freedom) was 0.015, implying that mean LDL-C may vary by country and statin intensity and that the effect of statin intensity may differ across the countries. In each country, LDL-C levels were higher among patients not treated with statins (Fig. 3). There was no consistent trend in LDL-C across patients with different eGFR levels (Fig. 4 and Supplemental Fig. 2; see file Supplementary Fig. 2). German patients had higher LDL-C levels, and US patients had lower LDL-C levels across all eGFR levels independent of serum albumin levels. These observations did not vary by the status of comorbidities such as ASCVD, diabetes, or peripheral artery disease (Supplemental Fig. 4; see file Supplementary Fig. 3).

## Discussion

In the present study, although there were no major differences in demographic and clinic characteristics between countries, there was a substantial variation in dyslipidemia management across geographies. A similar pattern of LLT underutilization across countries and CKD stages was observed, and different statins (high *vs.* low intensity) were prescribed in each country. In this group of CKD patients under nephrology care, LDL-C was lower among treated patients and differed significantly by country, with the highest LDL-C levels detected in Germany and the lowest in the US. Also, statin use was higher among patients over 50 and in patients with CV comorbidities or diabetes. These findings reflect a low adherence to evidence-based recommendations in real-world nephrological practice. It is important to mention that, in part, this low-adherence may be a result of the differences in access to treatment due to insurance coverage of medications, particularly in the US.

At the patient level, LDL-C did not vary significantly by CKD stage, regardless of statin use. LDL-C levels were also consistently higher for non-statin users in each country (+ 22 mg/dL overall in adjusted models), while low-intensity statin users had higher LDL-C levels than high-intensity statin users (+ 4 mg/dL overall in adjusted models). Different results were also demonstrated in the literature. The Pravastatin Pooling Project demonstrated pravastatin, in our study considered as a low-intensity statin, reduced LDL-C levels by 47.9 ± 24.1 mg/dL and triglyceride levels by 17.3 ± 56.3 mg/dL and raised HDL cholesterol by 2.3 ± 6.0 mg/dL at 12 months [9]. On the other hand, in the *Justification for the Use of Statins in Prevention: An Intervention Trial Evaluating Rosuvastatin (JUPITER),* rosuvastatin (high-intensity statin) reduced LDL-C by 52% [10].

It is important to remember that patients with moderate to advanced CKD present a mixed dyslipidemia pattern, with a combination of hypertriglyceridemia, low levels of HDL cholesterol, and variable levels of LDL cholesterol and total cholesterol [11–13]. In general, the progression of CKD to later stages impacts the lipid profile’s composition, resulting in a more atherogenic profile [11, 14]. Because of the combination of this atherogenic dyslipidemia profile with multiple comorbidities and extremely high CV risk, cardiology guidelines tend to recommend an aggressive and inclusive treatment regimen with statins in patients with CKD not on dialysis. However, all dialysis studies failed to demonstrate a significant reduction in cardiovascular events or mortality with LLT despite significant LDL cholesterol lowering, regardless of different treatment strategies applied [8, 15–18]. In contrast, the overall and non-dialysis subgroup analysis of the SHARP trial supports the aggressive treatment for CKD non-dialysis patients to reduce CV events.

Recently, the International Study of Comparative Health Effectiveness with Medical and Invasive Approaches (ISCHEMIA) and International Study of Comparative Health Effectiveness with Medical and Invasive Approaches—Chronic Kidney Disease (ISCHEMIA-CKD) trials found similar effects in reducing all-cause death or myocardial infarction between initial invasive management compared to initial conservative management, which includes aggressive LDL-C therapies as a standard of care, of patients with chronic coronary disease and moderate to severe ischemia on stress testing without or with advanced CKD [19, 20]. Also, a post-hoc analysis of the Aggressive Lipid-Lowering Initiation Abates New Cardiac Events (ALLIANCE) Study demonstrated that in patients with coronary heart disease and CKD, intensive LLT with higher doses of atorvastatin to achieve a target < 80 mg/dL reduced the relative risk of time to the first cardiovascular event by 28% in patients with CKD (HR, 0.72; 95% CI, 0.54 to 0.97; *P* = 0.02) and 11% in patients without CKD (HR, 0.89; 95% CI, 0.74 to 1.07; *P* = 0.3) [21]. In combination, these studies provide additional evidence to the potential benefits of LLT in CKD patients and raises the question: should nephrologists adopt the guidelines from other areas and redefine triggers and targets for LLT?

On the other hand, the CKD-REIN study found that lipid goal achievement was not associated with risk of fatal/non-fatal atheromatous CVD or non-atheromatous CVD in patients with non-dialysis CKD [8]. Safety considerations may also temper the use of intensive/high-dose LLT in patients with CKD, as statin-related toxicity is dose-related. An observational study in the general population demonstrated that in comparison with atorvastatin, rosuvastatin was associated with an increased risk of hematuria (HR, 1.08; 95% confidence interval [95% CI], 1.04 to 1.11), proteinuria (HR, 1.17; 95% CI, 1.10 to 1.25), and kidney failure with replacement therapy (HR, 1.15; 95% CI, 1.02 to 1.30) [22]. The risk was higher with a higher rosuvastatin dose [22]. Among patients with eGFR < 30 ml/min per 1.73 m^2^, 44% were prescribed daily high dose rosuvastatin (20 or 40 mg daily), which exceeds the FDA’s recommended 10 mg daily dose. These findings suggest the need for caution in prescribing and monitoring rosuvastatin, especially in patients receiving high doses or who have severe CKD. In this case, the difference in doses of statin LLT seems to influence patient outcomes [8, 22]. Another important point to be considered regarding safety, is the potential statins have of inducing rhabdomyolysis [23], because in patients with CKD it is important to avoid measures that could exacerbate kidney disfunction. Individualized treatment is a keystone to minimize those safety issues and improve outcomes in our patients.

Our study demonstrated that a substantial proportion of hyperlipidemic CKD patients are not receiving LLT, and 7–23% of untreated patients in each country had LDL-C ≥ 160 mg/dL. The kidney-focused KDIGO guidelines recommend no lipid goals or follow-up lipid testing, instead espousing a ‘fire-and-forget’ strategy of fixed-dose, moderate-intensity statin, or statin-ezetimibe therapy [4]. In contrast, the American Heart Association/American College of Cardiology (AHA/ACC) guidelines utilize LDL-based and ASCVD-risk-based thresholds for statin therapy among CKD patients but also do not stipulate treatment goals; they recommend a moderate-intensity statin alone or combined with ezetimibe for adults 40–75 years of age with LDL-C 70–189 mg/dL (1.7–4.8 mmol/L) who are at 10-year atherosclerotic CVD risk of ≥ 7.5% [24]. Conversely, European and UK guidelines recommends treat-to-goals strategies. For example, the European Society of Cardiology/European Atherosclerosis Society (ESC/EAS) guidelines distinguish patients with G3a-3b as high risk, and eGFR G4-5ND as very high risk, with a treatment goal of ≥ 50% LDL-C reduction from baseline and an LDL-C goal of < 1.8 mmol/L (< 70 mg/dL) for high-risk patients, and a goal of < 1.4 mmol/L (< 55 mg/dL) for very high-risk patients [5]. The UK Renal Association guidelines recommend goals for total cholesterol (≤ 4 mmol/L), LDL (≤ 2 mmol/L), and non-HDL (≤ 2.5 mmol/L) [25].

Our CKDopps nephrologist survey demonstrated the most common LDL-C goal was < 100 mg/dL across regions, regardless of CKD stage, but only 8–19% of nephrologists would aim for < 70 mg/dL for G4-5 patients, as per the current ESC guidelines for ‘very high risk’ patients. LDL-C levels did not appear to vary with eGFR in our analysis. The CKD-REIN study previously demonstrated that only 45% of ‘high-risk’ patients and 29–38% of patients at ‘very high risk’ achieved the LDL-C goal, based on the 2016 ESC/EAS guidelines, which recommended less aggressive targets than the current guidelines [26]. In the current study, overall statin use ranged from 51–61% of patients in countries included in this analysis. These findings are similar to a recent Canadian cross-sectional study of CKD patients, in which approximately 63% of statin-eligible patients were taking a statin [27]. This study also demonstrated CKD patients had about five times the odds of receiving statin therapy for secondary *vs.* primary prevention. Patients planning for conservative care had lower odds of being prescribed a statin than patients planning for dialysis [27]. There are several possible reasons why nephrologists may not prescribe statins for CKD patients, particularly for primary prevention. Respondents to a Canadian nephrologist survey cited some reasons, including disagreement with KDIGO guidelines in favor of a patient-individualized approach that considers life expectancy and the cause of CKD [27]. For example, for older patients with limited life expectancy, some nephrologists were concerned with the lack of evidence of benefit with statins, higher risk of adverse effects, and increased pill burden. The AHA/ACC does indicate it may be reasonable to stop statin therapy among patients > 75 years of age who have functional decline, multimorbidity, frailty, or reduced life expectancy [24]. While the SHARP trial did show evidence of a benefit for statin plus ezetimibe among the subgroup of CKD patients > 70 years of age (risk ratio 0.78, 95% CI 0.65–0.89) [15], there remains uncertainty about the benefit-risk ratio of statin use for primary prevention in people > 70 years of age in the general population.

Our study has some limitations. First, there is some heterogeneity in study protocols. For example, in France, lab data were drawn according to a country-specific protocol and, therefore, not included in the analysis of lipid monitoring which may compromise the results’ generalizability. Another limitation of this study is the absence of statin dose data, which made necessary an adjustment on the statin intensity classification compared to what is suggested by the guidelines. On the other hand, this study’s strengths are that this is a large, international cohort in academic and community settings and, therefore, may reflect the real-world dyslipidemia management in CKD patients under nephrology care.

In conclusion, there is substantial variation in practice patterns regarding lipid-lowering therapies across countries but not across CKD stages. Patients on LLT have lower LDL-C, yet in contrast to the evidence-based guideline recommendations, a significant proportion of CKD patients with dyslipidemia managed by nephrologists are not receiving LLT. Despite the literature supporting LLT in the CKD population with care in dose prescription, our study suggests that the nephrology community needs to review the recommendations and improve the implementation of these guidelines in clinical practice, which may lead to a reduction in the burden of CVD in the CKD population. Further comparative effectiveness studies assessing the effects of a ‘fire-and-forget’ strategy *vs.* a treat-to-target strategy on patient outcomes are needed to inform the optimal approach to lipid management in patients with non-dialysis CKD.

## Supplementary Information

**Additional file 1: Supplementary Figure 1. **Prevalence and intensity of statin use by country and other patient strata. Atorvastatin and rosuvastatin are categorized as high intensity; all other statins are categorized as low intensity: simvastatin, lovastatin, pravastatin, fluvastatin, cerivastatin, and pitavastatin.

**Additional file 2: Supplementary Figure 2.** Mean LDL-C during CKD progression, by country and other patient strata.

**Additional file 3: Supplementary Figure 3.** Distribution of LDL-C (mg/dL) by statin use and by other patient strata.

**Additional file 4: Supplementary Figure 4.** Adjusted mean LDL-C (mg/dL) by country and statin use. Legend: LDL-C levels adjusted to average age, sex, CKD stage, and comorbid risk status through a linear regression model.

## Data Availability

The datasets used and/or analysed during the current study are available from the corresponding author on reasonable request.

## Abbreviations

AHA/ACC: American Heart Association/American College of Cardiology
ASCVD: Atherosclerotic CVD
CKD: Chronic kidney disease
CKDopps: Chronic Kidney Disease Outcomes and Practice Patterns Study
CV: Cardiovascular
CVD: Cardiovascular disease
ESC/EAS: European Society of Cardiology/European Atherosclerosis Society
KDIGO: Kidney Disease Improving Global Outcomes
LDL-C: LDL- cholesterol
LLT: Lipid-lowering therapy
