# Supplementary figures and images for: International practice patterns of dyslipidemia management in patients with chronic kidney disease under nephrology care: is it time to review guideline recommendations?
Source: Lipids Health Dis. 2023 May 25;22:67. doi: 10.1186/s12944-023-01833-z (PMC10210460; doi:10.1186/s12944-023-01833-z)

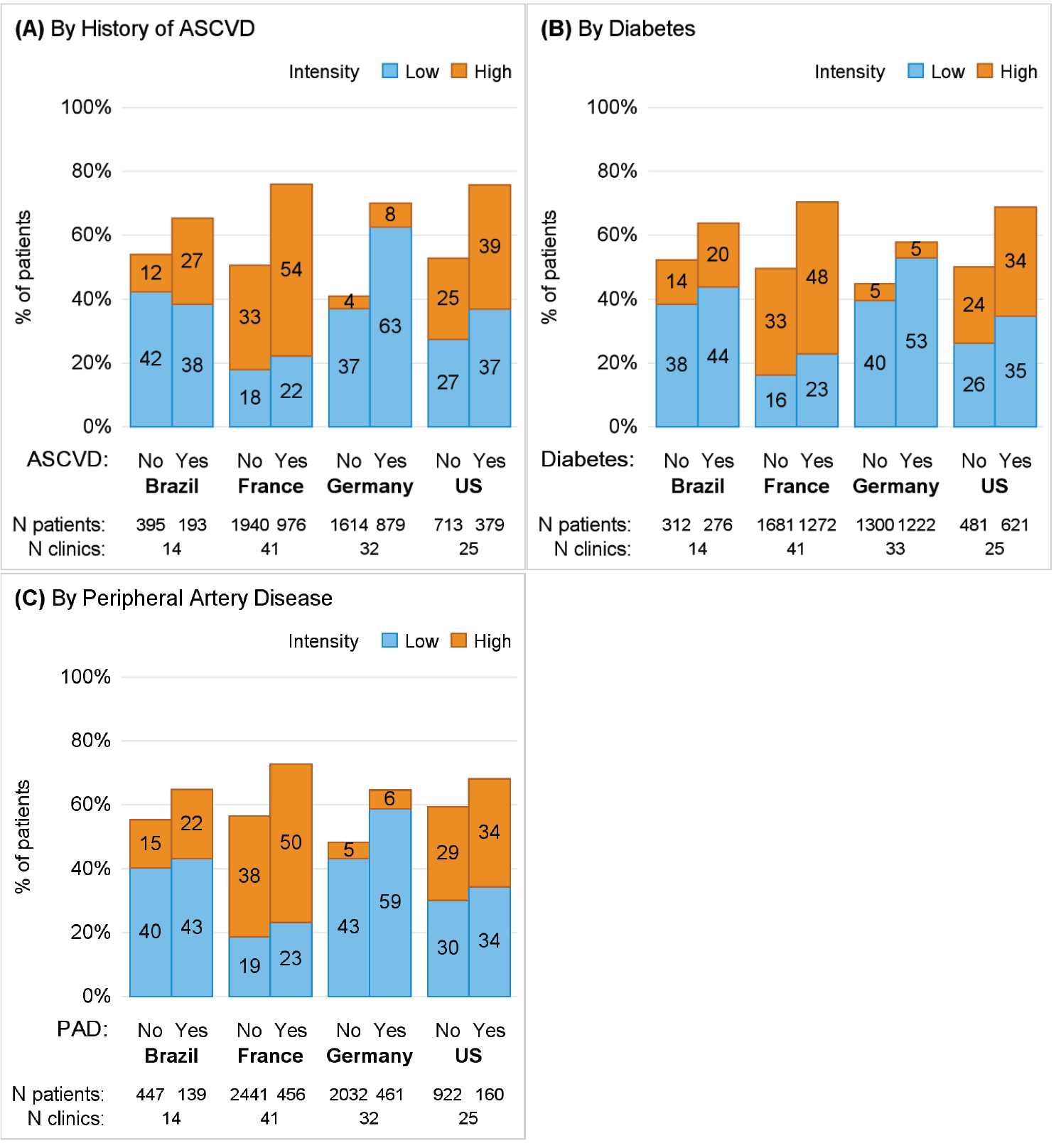

Supplement: Supplementary file 1 — Additional file 1: Supplementary Figure 1. Prevalence and intensity of statin use by country and other patient strata. Atorvastatin and rosuvastatin are categorized as high intensity; all other statins are categorized as low intensity: simvastatin, lovastatin, pravastatin, fluvastatin, cerivastatin, and pitavastatin. [file 12944_2023_1833_MOESM1_ESM.tif]

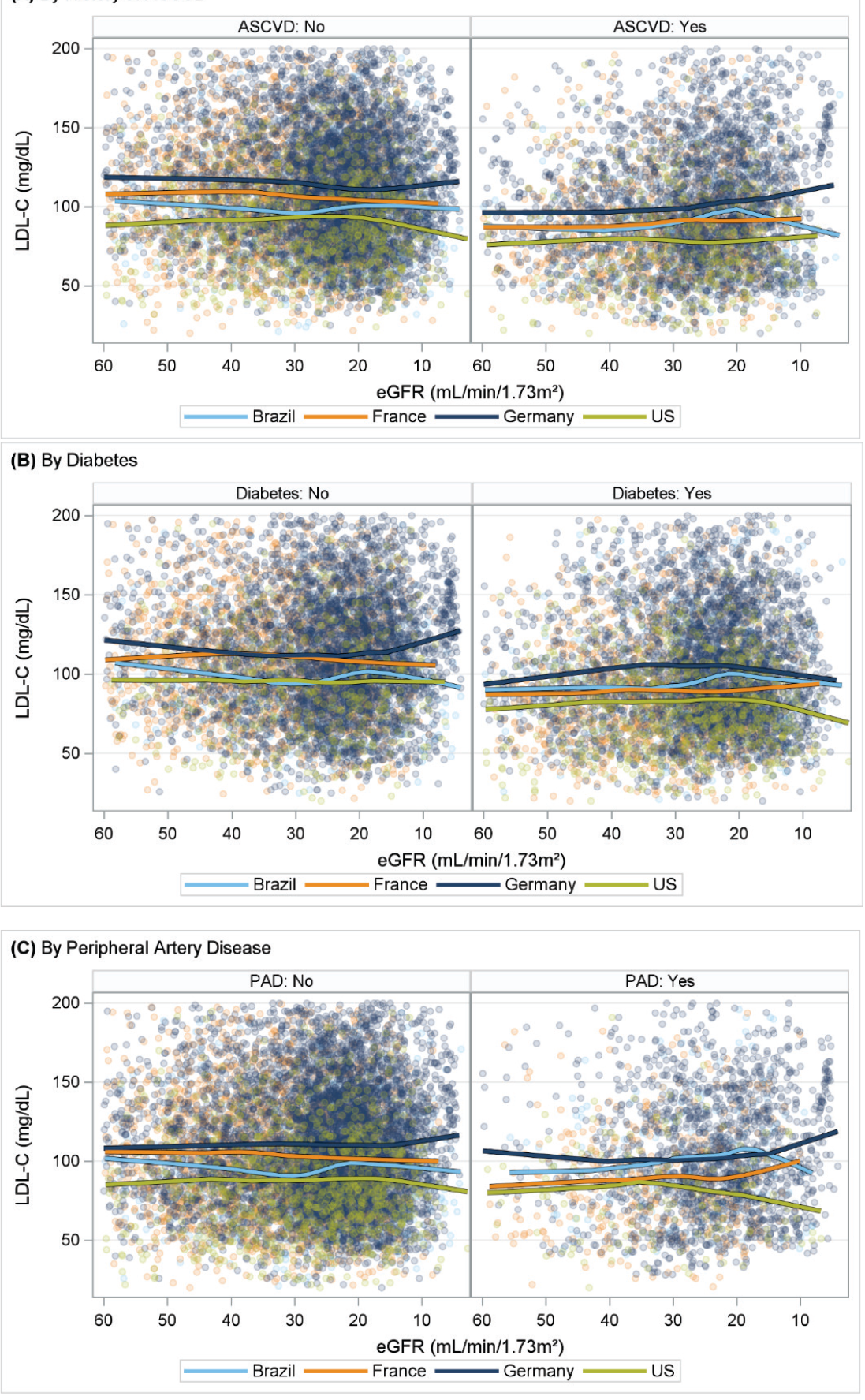

Supplement: Supplementary file 2 — Additional file 2: Supplementary Figure 2. Mean LDL-C during CKD progression, by country and other patient strata. [file 12944_2023_1833_MOESM2_ESM.tif]

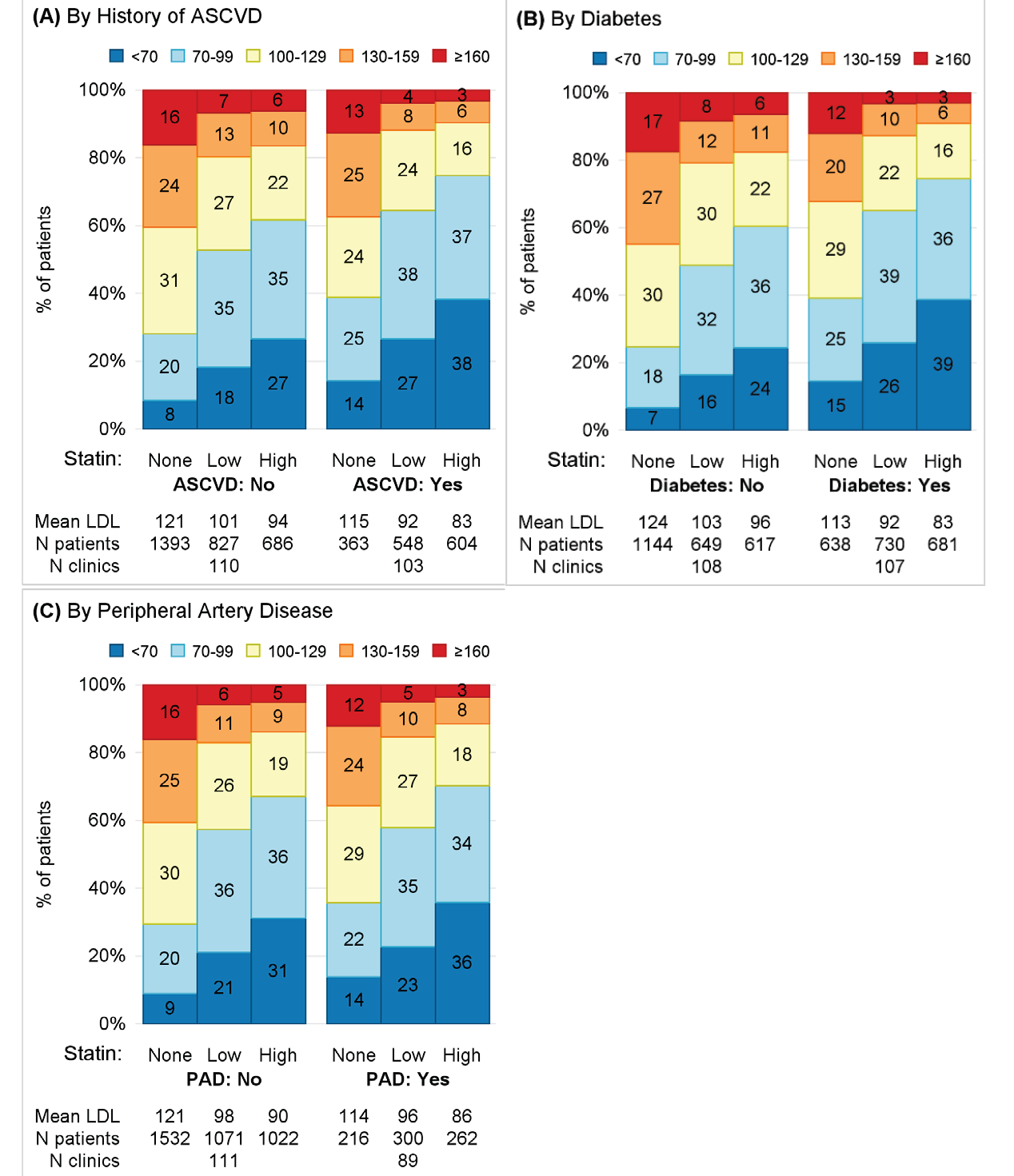

Supplement: Supplementary file 3 — Additional file 3: Supplementary Figure 3. Distribution of LDL-C (mg/dL) by statin use and by other patient strata. [file 12944_2023_1833_MOESM3_ESM.tif]

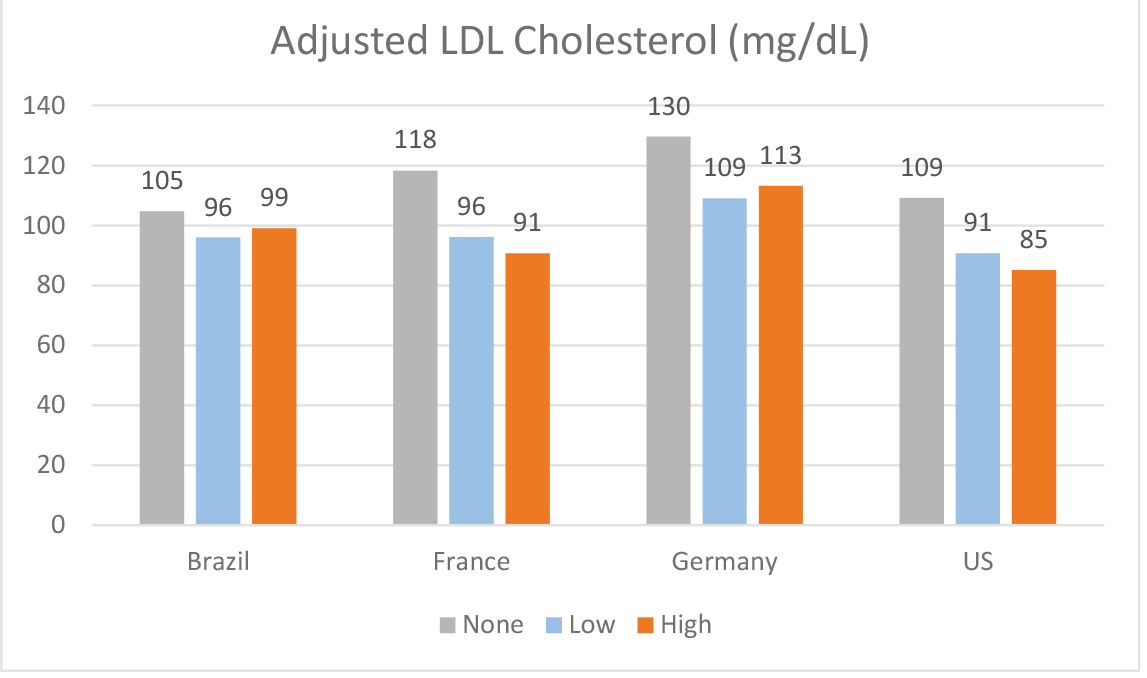

Supplement: Supplementary file 4 — Additional file 4: Supplementary Figure 4. Adjusted mean LDL-C (mg/dL) by country and statin use. Legend: LDL-C levels adjusted to average age, sex, CKD stage, and comorbid risk status through a linear regression model. [file 12944_2023_1833_MOESM4_ESM.tif]
